# Mining imaging and clinical data with machine learning approaches for the diagnosis and early detection of Parkinson’s disease

**DOI:** 10.1038/s41531-021-00266-8

**Published:** 2022-01-21

**Authors:** Jing Zhang

**Affiliations:** Department of Neurology, School of Medicine, Washington University in St. Louis, St. Louis, MO 63110 USA

**Keywords:** Parkinson's disease, Translational research

## Abstract

Parkinson’s disease (PD) is a common, progressive, and currently incurable neurodegenerative movement disorder. The diagnosis of PD is challenging, especially in the differential diagnosis of parkinsonism and in early PD detection. Due to the advantages of machine learning such as learning complex data patterns and making inferences for individuals, machine-learning techniques have been increasingly applied to the diagnosis of PD, and have shown some promising results. Machine-learning-based imaging applications have made it possible to help differentiate parkinsonism and detect PD at early stages automatically in a number of neuroimaging studies. Comparative studies have shown that machine-learning-based SPECT image analysis applications in PD have outperformed conventional semi-quantitative analysis in detecting PD-associated dopaminergic degeneration, performed comparably well as experts’ visual inspection, and helped improve PD diagnostic accuracy of radiologists. Using combined multi-modal (imaging and clinical) data in these applications may further enhance PD diagnosis and early detection. To integrate machine-learning-based diagnostic applications into clinical systems, further validation and optimization of these applications are needed to make them accurate and reliable. It is anticipated that machine-learning techniques will further help improve differential diagnosis of parkinsonism and early detection of PD, which may reduce the error rate of PD diagnosis and help detect PD at pre-motor stage to make it possible for early treatments (e.g., neuroprotective treatment) to slow down PD progression, prevent severe motor symptoms from emerging, and relieve patients from suffering.

## Introduction

Parkinson’s disease (PD) is a common, chronic, progressive neurodegenerative movement disorder associated with the aggregation of abnormal α-synuclein in Lewy bodies and the loss of nigrostariatal dopaminergic neurons. The mechanism of neurodegeneration in PD is unclear, and currently, there is no cure for PD. The most striking symptoms of PD are motor symptoms such as tremor, rigidity, bradykinesia, or postural instability and patients with severe motor symptoms often have difficulty using their hands, or have difficulty standing and walking due to tremor and stiff muscles, which severely affects their quality of life. In addition, non-motor symptoms, such as hyposmia/anosmia (smell/olfactory loss), autonomic dysfunction, and rapid eye movement (REM) sleep behavior disorder, usually emerge years before motor symptoms, but they may be mild and are often overlooked. The diagnosis of PD is challenging, e.g., in differentiating PD from essential tremor, drug-induced parkinsonism and atypical parkinsonian disorders such as progressive supranuclear palsy (PSP), multiple system atrophy (MSA), and corticobasal degeneration (CBD). The error rate of a clinical diagnosis of PD is high. A meta-analysis reported that the error rate was 26.2% by nonexperts, and from 16.1% (for initial diagnosis) to 20.4% (for follow up diagnosis) by experts^1^. Using autopsy results to evaluate PD diagnoses, Hughes et al.^2^ found the diagnostic error rate was around 24%. Further, using neuropathologic findings of PD as the gold standard, Adler et al.^3^ found that the accuracy of a clinical diagnosis of PD was only 26% in untreated or medication non-responsive subjects, 53% in medication-responsive early PD (duration shorter than 5 years), and >85% in medication-responsive and longer duration PD. The high error rate in PD diagnosis may be because: (1) Clinical diagnoses of PD are mainly based on results of clinical tests and response to antiparkinsonian medication. Neuroimaging is only used as an assistance in PD diagnosis, although the clinical utility of neuroimaging such as SPECT (single photon emission computed tomography) is high and results of dopamine transporter scan (DaTscan) lead to modified diagnosis in one-third of the patients^4^; (2) Currently, there are few reliable biomarkers for PD^5^, in particular, there is no in vivo imaging tool available to directly image the accumulation of α–synuclein aggregates or the spreading of Lewy bodies in the brain of a PD patient^6^.

Another challenge in the diagnosis of PD is early detection because at early stages of PD, brain changes and symptoms are subtle. The brain regions that are most affected by PD are the basal ganglia and substantia nigra. Neurodegeneration of the basal ganglia and loss of dopaminergic neurons in the substantia nigra begin long before the presence of motor symptoms, and by the time motor symptoms emerge, 40–60% of nigral dopaminergic neurons are lost and up to 80% synaptic function is reduced^7,8^. The period between the onset of neurodegeneration and the emergence of motor symptoms is called prodromal (or pre-motor) stage, which might last from several years to decades^9^. Early neuroprotective treatment can slow down neurodegeneration progression and potentially prevent clinical PD symptoms from emerging^9^. Therefore, it is important to detect PD at early stages so that early neuroprotective treatment can be effective.

Clinical assessment and analysis of PD imaging is crucial for the diagnosis of PD. At early stages of PD, loss of neurons in the brain first occurs in the ventrolateral substantia nigra pars compacta, then projects to the posterior putamen, and then to more regions in the striatum. Progressive brain atrophy has been detected on structural MRI in PD, even at early PD stages^10,11^. In addition, due to the accumulation of abnormal α-synuclein aggregates in Lewy bodies and the spreading of Lewy bodies in the brain of PD patients over time (from brain stem and olfactory system, to the substantia nigra and then to neocortical regions)^12^, PD may be viewed as a progressive brain network disruption^13^. Dopaminergic radiotracer imaging with SPECT or positron emission tomography (PET), a biomarker of early PD, can detect dopaminergic denervation in PD at pre-motor stage^9,14^. However, dopaminergic denervation may elude from visual analysis or semi-quantitative image analysis of SPECT or PET images. Consequently, there is variability in dopamine transporter SPECT imaging interpretation between radiologists, which leads to inconsistent diagnosis. Thus, computer-aided diagnosis (CAD) based on machine-learning methods has been developed to help detect dopaminergic denervation on SPECT^15–20^ and PET^21–24^ images, and to identify PD-related structural changes on MRI^25–28^ for early detection of PD. Moreover, resting-state functional MRI (rs-fMRI)^29–33^ and diffusion tenser imaging (DTI)^34,35^ have been used to identify abnormal functional and structural connectivity in PD. Further, machine-learning-based multi-modal data (including imaging and/or clinical data) analysis has been found helpful in the detection of brain abnormalities in PD^17,30,36,37^.

Machine learning (ML), a group of multivariate analytic methods that learn from data, identify data patterns and classify the data, is often used in data mining and artificial intelligence. Machine learning can be either supervised (using training data labeled by humans for data classification) or unsupervised (which does not use training data, but identify data patterns on its own). Supervised learning includes methods such as linear discriminant analysis (LDA)^38^, support vector machine (SVM)^39^, artificial neural networks (ANNs)^40^ and random forest^41^, while unsupervised learning includes approaches such as cluster analysis^42^. Among these methods, SVM and ANNs are frequently used machine-learning models. SVM creates a line (or a hyperplane) that best separates data into classes and provides linear (or non-linear) mapping between inputs and outputs, while ANNs (consisting of multiple layers) work in a complex and non-linear way, which do not provide direct mapping between inputs and outputs. In addition, based on ANNs, newly developed deep-learning techniques or deep-neural networks are a new set of powerful tools for data classification in PD^18,24,43^.

Since each PD case is unique, diagnosis and therapy need to be tailored for individual patients to achieve the best clinical outcome. Machine-learning (ML) techniques have the potential to identify complex data patterns, automate data analysis, and make inferences/classifications for data of individual patients, which may be useful for precision medicine in PD. In recent years, machine learning has been increasingly used in the diagnosis of PD. This paper reviewed the studies that applied machine-learning methods to the diagnosis and early detection of PD in order to provide an overview of this field.

## An overview of machine-learning-based studies for the diagnosis of PD

Studies applied machine-learning-based approaches to the diagnosis of PD mainly fall into three categories: 1. Discrimination between PD and Healthy control (HC); 2. Differential diagnosis; 3. Early PD detection. Machine-learning-based imaging studies using SPECT, PET, structural MRI, and functional MRI (fMRI) were summarized in Table 1, Table 2, Table 3, and Table 4, respectively (among them, Table 4 is a Supplementary Table).

**Table 1:** Machine-learning-based SPECT dopaminergic imaging studies for PD diagnosis and early detection.

Study	Sample	Data features	Methods	Main findings	Other findings
Acton and Newberg, 2006^15^	81 PD, 94 HC	Striatum images of from [99mTc]TRODAT-1 SPECT images	Feature selection: Down-sample voxels in the striatum; Classification: ANN; Validation: Leave-one-out	Classification accuracy: 94.4%	ANN performed better than semi-quantitative ROI analysis (81.3%) and radiologists (88%); Difficult to interpret what ANN detect in the image
Hamilton et al., 2006^70^	18 PD (12 advanced PD, 6 early PD with ET)	Striatal uptake ratios (striatum-to-occipital cortex ratio and putamen-to-caudate tracer accumulation ratio) from 123I-FP-CIT SPECT images	Feature selection: none; Classification: ANN; Validation: Leave-one-out	Classification accuracy: 100%	Putamen-to-caudate tracer accumulation ratio is able to discriminate between PD and ET
Palumbo et al., 2010^71^	261 PD (89 ET, 64 early PD, 63 advanced PD)	Striatal uptake and uptake ratios (putamen/occipital, caudate/occipital) from (123)I-FP-CIT SPECT images	Feature selection: Down-sample voxels in the striatum; Classification: PNN and CIT; Validation: 50-fold-cross-validation	Classification accuracy: For PNN: Early PD: 81.9 ± 8.1%; Advanced PD: 78.9 ± 8.1%; ET: 96.6 ± 2.6%	CIT provided reliable cut-off values (e.g., 5.99 at putamen and 6.97 at caudate); Classification accuracy: For CIT: Early PD: 69.8 ± 5.3%; Advanced PD: 88.1%±8.8%; ET: 93.5 ± 3.4%
Illan et al., 2012^44^	108 PD, 100 HC	Striatal uptake image with normalized high intensity from 123I-ioflupane SPECT images	Feature selection: Applied a mask for high-intensity voxels; Classification: SVM, KNN, NM; Validation: Leave-one-third-out	Classification accuracy: AUC: 0.968 for SVM; 0.931 for KNN; 0.942 for NM	Classifier selection had higher impact on classification results than the preprocessing steps; Image preprocessing with voxel intensity normalized to a maximum value performed the best
Segovia et al., 2012^45^	95 PD, 94 HC	Striatal uptake with high intensity from 123I-ioflupane SPECT images	Feature selection: PLS and down-sampling; Classification: SVM; Validation: Leave-one-out	Classification accuracy: 94.7% (AUC: 0.968)	PLS + SVM outperformed previous approaches based on singular value decomposition
Martinez-Murcia et al., 2014^47^	158 PD, 111 HC	Computed Haralick texture features (via a gray-level co-occurrence matrix) from 123I-ioflupane SPECT images	Feature selection: none; Classification: SVM; Validation: Leave-one-out	Classification accuracy: 97.4%	
Palumbo et al., 2014^46^	56 PD, 34 non-PD	Uptake in the caudate (CL, CR) and putamen (PL, PR) from 123I-FP-CIT SPECT	Feature selection: none; Classification: SVM; Validation: Leave-one-out; fivefold cross-validation	Classification accuracy: CL + CR + PL + PR: 90.6% (leave-one-out); 90.7% (fivefold cross-validation)	Adding age improved classification accuracy (95.6%)
Prashanth et al., 2014^16^	369 early PD, 179 HC (from PPMI)	Striatal binding ratios from 123I-Ioflupane SPECT images	Feature selection: none; Classification: SVM; Validation: tenfold cross-validation	Classification accuracy: 96.14 ± 1.89%	SVM with non-linear kernel of radial basis function achieved higher classification accuracy than SVM with linear kernel (92%)
Oliveira and Castelo-Branco, 2015^87^	445 early PD, 209 HC (from PPMI)	Binding potential at each voxel in the striatum as feature extracted from 123I-ioflupane SPECT images	Feature selection: Apply BP threshold; Classification: SVM; Validation: Leave-one-out	Classification accuracy: 97.86%	The classification results were robust regardless the reference VOI used or the transformation used (for spatial normalization), or the way the features selected
Hirschauer et al., 2015^37^	189 PD, 62 SWEDD, 415 HC (from PPMI)	Motor and non-motor clinical features such as motor function and olfactory loss and imaging biomarkers such as ioflupane (123I) SPECT striatal-binding ratios	Feature selection: none; Classification: EPNN, PNN, SVM, KNN and CT; Validation: tenfold cross-validation	Classification accuracy: PD vs. HC: 98.6% (EPNN), 98.2% (KNN), 98.1% (SVM); PD vs. SWEDD: 95.3% (EPNN), 94.6% (KNN), 89.3% (SVM)	Classification accuracy: SWEDD vs. HC: 92% (EPNN), 91.6% (KNN), 89.3% (SVM)
Huertas-Fernández et al., 2015^66^	80 VP, 164 PD	Uptake in the striatum and whole brain from 123I-ioflupane SPECT images	Feature selection: *t*-test and Mann–Whitney test for the important features; Classification: LR, LDA, SVM; Validation: tenfold cross-validation	Classification accuracy: 90.3 ± 5.8% (LR for ROI approach); 90.4 ± 5.9% (SVM for voxel-based whole-brain approach)	Classification accuracy: 89.8 ± 6.5% (LDA for ROI), 89.9 ± 4.9% (SVM for ROI); 88.7 ± 4.9% (LR for voxel-based) 88.4 ± 6.4% (LDA for voxel-based)
Prashanth et al., 2016^17^	401 early PD, 183 HC (from PPMI)	Non-motor clinical features such as RBD and olfactory loss, CSF measurements and SPECT imaging markers (striatal-binding ratios)	Feature selection: none; Classification: SVM, random forests; Validation: tenfold cross-validation	Classification accuracy: 96.40 ± 1.08% for SVM, 96.18 ± 1.27% for random forests	SVM outperformed other classifiers; Combined features (non-motor clinical features and CSF and imaging markers) are useful for early detection of PD
Choi et al., 2017^18^	431 PD, 77 SWEDD, 193 HC (from PPMI); SNUH data: 72 PD, 10 HC	Striatal binding ratios and other imaging features from SPECT images	Feature selection: none; Classification: Deep CNN; Validation: tenfold cross-validation	Classification accuracy: 96% (PPMI); 98.8% (SNUH)	The performance of PD Net (deep CNN) was comparable to that of experts; SWEDD could be reclassified by PD Net
Prashanth et al., 2017^88^	427 early PD, 80 SWEDD, 208 HC (from PPMI)	Shape and surface-fitting-based features, striatal-binding ratios from SPECT images	Feature selection: Estimate feature importance with random forest; Classification: SVM, random forests; Validation: tenfold cross-validation	Classification accuracy (early PD vs. non-PD (SWEDD/HC)): 97.29 ± 0.11% for SVM, 96.9 ± 0.17% for random forests	SVM outperformed other classifiers; Shape and surface-fitting-based features showed higher importance than striatal-binding ratios for classification
Wang et al., 2017^48^	369 PD,165 NC (from PPMI). [93 AD, 202 MCI, 101 HC (from ADNI)]	PPMI: Striatal blinding ratios from SPECT images; Gray matter, white matter, and CSF volumes of ROIs from MRI images; ADNI: Gray matter volume of the ROIs from MRI images; mean intensity of ROIs from PET images	Feature selection: Optimization in progressive transductive learning; Classification: SVM, GTL; Validation: tenfold cross-validation	Classification accuracy: PPMI (PD vs. HC): SVM: 88.5% (MRI + SPECT); GTL: 97.4% (MRI + SPECT); ADNI (AD vs. HC): SVM: 86.7 ± 1.42% (MRI + PET); GTL: 92.6 ± 0.65% (MRI + PET)	Multi-modal features led to better classification performance than single-modal features
Zhang and Kagen, 2017^49^	1171 PD, 131 SWEDD, 211 HC (from PPMI)	A slice that has the highest striatal signal-to-background ratio of SPECT image was used	Feature selection: gradient descent optimization; Classification: Artificial Neural network; Validation: tenfold cross-validation	Classification accuracy: PD vs. HC: 93.8 ± 4.7%	A comparison of gradient descent and the Adagrad optimizer showed that there was no significant difference in their classification performance
Taylor and Fenner, 2017^19^	113 non-PDD, 191 PDD (Local data); 448 PD, 209 HC (from PPMI)	Voxel intensities; Principal components of image voxel intensities; Striatal binding radios (from the putamen and caudate) from (I123) Ioflupane (FP-CIT) SPECT images	Feature selection (data dimension reduction): PCA; Classification: SVM; Validation: tenfold cross-validation	Classification accuracy: semi-quantitative methods: 78~87% (local data), 89 ~95% (PPMI); SVM: 88~92% (local data), 95 ~97% (PPMI)	Machine-learning method performed better than semi-quantitative methods
Taylor et al., 2018^20^	304 PD (113 without PDD, 191 with PDD); 448 PD, 209 HC (from PPMI)	First five principal components of image voxel intensities in the striatum extracted from (123I)FP-CIT SPECT images	Feature selection: none; Classification: SVM; Validation: tenfold cross-validation	Classification accuracy: 92%	CADx increased the accuracy of the radiologists for research images, but had no significant change in accuracy for the clinical data and had less impact on the clinical scientist
Oliveira et al., 2018^89^	443 early PD, 209 HC (from PPMI)	Striatum uptake ratios and striatum dimensional-based features extracted from (123I)FP-CIT SPECT images	Feature selection: none; Classification: SVM; KNN; LR; Validation: Leave-one-out cross-validation	Classification accuracy: 97.9% (SVM, all features)	SVM outperformed other classifiers such as KNN and LR; Features with high classification accuracy: the length of the striatal region (96.5%), the putaminal binding potential (95.4%) and the striatal-binding potential (93.9%)

*ADNI* Alzheimer’s disease neuroimaging initiative, *AUC* area under the ROC (receiver-operating characteristic) curve, *CADx* computer-aided diagnosis, *CIT* or *CT* classification tree, *CL* caudate left, *CNN* convolutional neural networks, *CR* caudate right, *CSF* cerebrospinal fluid, *EPNN* enhanced probabilistic neural network, *ET* essential tremor, *GLS-DBN* group Lasso sparse deep belief network, *GTL* graph-based transductive learning, *KNN* k-nearest neighbor, *LDA* linear discriminant analysis, *LR* logistic regression, *NM* nearest mean, *PCA* principal component analysis, *PD* Parkinson’s disease, *PDD* pre-synaptic dopaminergic deficit, *PL* putamen left, *PR* putamen right, *PLS* partial least squares, *PNN* probabilistic neural network, *PPMI* Parkinson’s progression markers initiative, *RBD* rapid eye movement (REM) sleep behavior disorder, *ROC* receiver-operating characteristic, *ROI* region of interest, *SVM* support vector machine, *SWEDD* scans without evidence of dopaminergic deficit, *VP* vascular parkinsonism.

**Table 2:** Machine-learning-based PET imaging studies for PD diagnosis and early detection.

Study	Sample	Data features	Methods	Main findings	Other findings
Segovia et al., 2015^67^	39 PD, 24 MSA, 24 PSP	Features of normalized intensity uptake values of the ROIs (putamen, thalamus, anterior cingulate gyrus, pars opercularis) from 18F-DMFP-PET images	Feature selection: 2-sample *t*-test for the importance of each ROI; Classification: SVM + Bayesian network; Validation: tenfold cross-validation	Classification accuracy: PD vs. non-PD: SVM + Bayesian network (4 ROIs): 78.16%	Classification accuracy: PD vs. non-PD: Using all voxels: 70.11%; Using ROIs only in the striatum: 73.56%; SVM (major voting): 74.71%; Multiple-kernel SVM: 75.86%
Segovia et al., 2017a^68^	39 PD, 24 MSA, 24 PSP	Features of normalized intensity values of the ROIs in the caudate, putamen, thalamus, olfactory, and SMA from 18F-DMFP-PET images	Feature selection: 2-sample *t*-test for the importance of each ROI; Classification: Multiple-kernel-learning SVM; Validation: tenfold cross-validation	Classification accuracy: PD vs. non-PD: 73.56% (using 5 ROIs)	Using 5 ROIs, classification accuracy was higher than that using 2 ROIs in the striatum (68.96%); and higher than that using DATSCAN (59.77%)
Segovia et al., 2017b^69^	39 PD, 24 MSA, 24 PSP	Features of normalized intensity values of the ROIs in the striatum, which was automatically segmented from 18F-DMFP-PET images	Feature selection: none; Classification: SVM; Validation: fivefold cross-validation	Classification accuracy: PD vs. non-PD: Stratum using automatic segmentation: 75.86%	Classification accuracy: Stratum using atlas: 72.41%; All voxels: 65.52%
Wang et al., 2017^48^	369 PD,165 NC (from PPMI). [93 AD, 202 MCI, 101 HC (from ADNI)]	PPMI: Striatal blinding ratios from SPECT images; Gray matter, white matter, and CSF volumes of ROIs from MRI images; ADNI: Gray matter volume of the ROIs from MRI images; mean intensity of ROIs from PET images	Feature selection: Optimization in progressive transductive learning; Classification: SVM, GTL; Validation: tenfold cross-validation	Classification accuracy: PPMI (PD vs. HC): SVM: 88.5% (MRI + SPECT); GTL: 97.4% (MRI + SPECT); ADNI (AD vs. HC): SVM: 86.7 ± 1.42% (MRI + PET); GTL: 92.6 ± 0.65% (MRI + PET)	Multi-modal features led to better classification performance than single-modal features
Glaab et al., 2019^21^	44~60 PD, 14~16 HC	Whole-brain uptake data extracted from FDOPA PET and FDG-PET; Metabolomics data from blood plasma	Classification: SVM, random forest; Validation: Leave-one-out	SVM AUC for FDOPA + blood metabolomics: 0.98; SVM AUC for FDG + blood metabolomics: 0.91	
Shen et al., 2019^22^	125 PD, 225 HC	Uptake data of stratum and other regions extracted from FDG PET	Classification: GLS-DBN Validation: Train-validation ratio: 80:20	Test set 1: Classification accuracy=90% (AUC = 0.912); Test set 2: Classification accuracy=86% (AUC = 0.899)	
Wu et al., 2019^23^	Cohort 1: 91 PD, 91 HC Cohort 2: 22 PD, 26 HC	Texture features of uptake data extracted from over 90 regions of interest on FDG PET using texture analysis	Classification: SVM Validation: fivefold cross-validation	Classification accuracy: Cohort 1: Accuracy = 91.26%; Cohort 2: Accuracy = 90.18%	
Zhao et al., 2019^24^	502 PD, 239 MSA, 179 PSP	Saliency features (using saliency maps of regions of interests) of uptake data extracted from FDG PET	Classification: CNN Validation: sixfold cross-validation	Classification accuracy: For PD: Sensitivity = 97.7%, Specificity = 94.1%; For MSA: Sensitivity = 96.8%, Specificity = 99.5%; For PSP: Sensitivity = 83.3%, Specificity = 98.3%	

*ADNI* Alzheimer’s disease neuroimaging initiative, *AUC* area under the ROC (receiver-operating characteristic) curve, *CADx* computer-aided diagnosis, *CIT* or *CT* classification tree, *CL* caudate left, *CNN* convolutional neural networks, *CR* caudate right, *CSF* cerebrospinal fluid, *EPNN* enhanced probabilistic neural network, *ET* essential tremor, *GLS-DBN* group Lasso sparse deep belief network, *GTL* graph-based transductive learning, *KNN* k-nearest neighbor, *LDA* linear discriminant analysis, *LR* logistic regression, *NM* nearest mean, *PCA* principal component analysis, *PD* Parkinson’s disease, *PDD* pre-synaptic dopaminergic deficit, *PL* putamen left, *PR* putamen right, *PLS* partial least squares, *PNN* probabilistic neural network, *PPMI* Parkinson’s progression markers initiative, *RBD* rapid eye movement (REM) sleep behavior disorder, *ROC* receiver-operating characteristic, *ROI* region of interest, *SVM* support vector machine, *SWEDD* scans without evidence of dopaminergic deficit, *VP* vascular parkinsonism.

**Table 3:** Machine-learning-based structural MRI studies for PD diagnosis and early detection.

Study	Sample	Data	Methods	Main findings	Other findings
Duchesne et al., 2009^75^	16 PD, 8 probable PPS, 8 probable MSA, 149 HC	Intensity and shape-based features for brain tissue composition and deformation in the hindbrain region from MRI	Feature selection: PCA; Classification: SVM with least-squares optimization; Validation: leave-one-out	Classification accuracy (PD vs. PSP or MSA): 91% (sensitivity 79~87%, specificity 87~96%)	Automatic imaging feature extraction and classification may aid in the diagnosis of PD vs. PSP or MSA
Focke et al., 2011b^76^	21 PD, 10 PSP, 11 MSA, 22 HC	GM and WM volume from MRI (by VBM)	Feature selection: threshold images; Classification: SVM; Validation: leave-one-out	Classification accuracy: (PD vs. PSP) 87.1% for GM 96.8% for WM; (PD vs. MSA) 71.9% for GM 65.63% for WM	GM and WM volume did not differentiate PD from HC
Haller et al., 2012^84^	17 PD, 23 other Parkinsonism (5 MSA; 1 PSP; 17 other types)	TBSS from DTI	Feature selection: select the most discriminative features with RELIEF; Classification: SVM; Validation: tenfold cross-validation	Classification accuracy: 97.5 ± 7.54% (PD vs. other Parkinsonism)	PD had a spatially consistent increase in FA and decrease in MD in the right frontal white matter
Haller et al., 2013^78^	16 PD, 20 other Parkinsonism	SWI	Feature selection: select the most discriminative features with RELIEF; Classification: SVM; Validation: tenfold cross-validation	PD had increased SWI in the bilateral thalamus and left substantia nigra; Classification accuracy: 86.92 ± 16.59% (PD vs. Other)	Visual analysis yielded no differences between groups
Salvatore et al., 2014^77^	28 PD, 28 PSP, 28 HC	Imaging features obtained by PCA; Voxel-based pattern distribution map of structural differences from MRI	Feature selection: PCA; Classification: SVM; Validation: leave-one-out	Classification accuracy (Specificity/Sensitivity): 93.5 (90.6/97.4)% for PD vs HC; 92.2 (92.5/92.4)% for PSP vs HC; 92.2 (91.3/94.4)% for PSP vs PD	Regions in the midbrain, pons, corpus callosum and thalamus
Cherubini et al., 2014^85^	57 probable PD, 21 PSP (9 with probable PSP and 12 with possible PSP)	GM and WM volumes from MRI; FA and MD from DTI; DAT-SPECT used as ground truth	Feature selection: *F*-test for the important ROIs; Classification: SVM; Validation: Leave-one-out	Classification accuracy: All features combined: 100%; GM + MD + FA: Sensitivity: 90%; Specificity: 96%	Classification accuracy: Sensitivity: 76% (GM), 100% (WM), 86% (FA), 57% (MD); Specificity: 93% (GM), 100% (WM), 88% (FA), 93% (MD)
Singh and Samavedham, 2015^26^	518 early PD, 68 SWEDD, 245 HC (from PPMI)	Voxel intensity change images, and GM and WM volumes of 500 ROIs from MRI (by KSOM)	Feature selection: WAT; Classification: Least-squares SVM; Validation: 20-fold cross-validation	Classification accuracy: PD vs. HC: 93.25 ± 0.46% for GM; 96.84 ± 0.28% for WM; PD vs. SWEDD: 99.86 ± 0.1% for GM 98.59 ± 0.48% for WM; SWEDD vs. HC: 100 ± 0% for GM 99.21 ± 0.36% for WM	Compared with HC, PD had atrophy in regions such as putamen, thalamus, and corpus callosum; Volume loss in regions such as cerebellum may help differentiate e SWEDD with PD
Dinov et al., 2016^36^	263 PD, 40 SWEDD, 127 HC (from PPMI)	Clinical data (e.g., UPDRS scores), demographic data (e.g., age), genetics data (e.g., chr12), and neuroimaging biomarker (e.g., cerebellum shape index) from MRI	Feature selection: hillclimbing search, CARET; Classification: Model-based such as GLM and MMRM; Data-driven: AdaBoost, SVM, Naïve Bayes, Decision Tree, KNN, K-Means; Validation: fivefold cross-validation	Classification accuracy: PD vs. HC: 96.2% for SVM, 98.9% for AdaBoost; PD + SWEDD vs. HC: 94.5% for SVM, 98.3% for AdaBoost	Model-free or data-driven methods outperformed model-based methods; Including UPDRS data improved classification accuracy
Huppertz et al., 2016^74^	204 PD, 73 HC, 106 PSP, 21 MSA	Volumetric measures (of 44 ROIs in GM, WM, CSF, brain lobes, cerebellum, midbrain, etc.) from MRI	Feature selection: none; Classification: SVM; Validation: Leave-one-out	Atrophy in the midbrain, basal ganglia, and cerebellar peduncles contributed most to classification; Classification accuracy: PD vs. HC: 66.2%; PSP vs. HC: 91.4%; MSA vs. HC: 82.4-88.4%; Multi-class classification: PD: 86.9%; PSP: 85.4%; MSA: 87.2%	Midbrain atrophy is the hallmark of PSP; Atrophy in pons is most prominent in MSA; PD had subtle volume reduction in cerebral GM (esp. basal ganglia)
Adeli et al., 2016^90^	374 PD, 169 HC (from PPMI)	GM, WM volumes of 98 ROIs from MRI	Feature selection: JFSS; Classification: Robust LDA; SVM; Validation: Leave-one-out	Classification accuracy: 81.9% for Robust LDA; 69.1% for SVM	Feature selection with JFSS and classification with robust LDA outperformed other feature selection and classification methods; This approach can be applied to other neurodegenerative disorders
Gu et al., 2016^80^	52 PD (19 PIGD, 25 TD, 8 mixed subtype), 45 HC	GM, WM, CSF volumes from MRI; FA, MD, RD, AD from DTI; ReHo and ALFF from resting-state fMRI	Feature selection: Recursive feature elimination; Classification: SVM; Validation: Leave-one-out	Classification accuracy: PIGD vs. non-PIGD 92.3%	The diagnostic agreement evaluated by the Kappa test showed Kappa value = 0.83 for agreement with the existing clinical categorization
Zeng et al., 2017^52^	45 probable PD, 40 HC	GM in the cerebellum from MRI	Feature selection: Recursive feature elimination; Classification: SVM; Validation: Leave-one-out; fivefold (twofold, 632-fold) cross-validation	Classification accuracy: 97.7% for leave-one-out validation, 97.2% and 96.9% for twofold and fivefold cross-validation respectively	PD had GM density decrease in the Crus and Vermis of the cerebellum
Du et al., 2017^86^	35 PD, 36 HC, 16 MSA, 19 PSP	DTI (FA, MD) and the R2* (apparent transverse relaxation rate) measures in the striatal, midbrain, limbic, and cerebellum	Feature selection: Regularized logistic regression; Classification: Elastic-Net machine learning and receiver-operating characteristic curve analysis; Validation: nested tenfold cross-validation	Classification accuracy: PD vs. HC: 91% (DTI + R2*), 82% (DTI); PD vs. MSA: 99% (DTI + R2*), 89% (DTI); PD vs. PSP: 99% (DTI + R2*), 97% (DTI); MSA vs. PSP: 98% (DTI + R2*), 96% (DTI);	MSA showed decreased FA and an increased R2* in the subthalamic nucleus, whereas PSP showed an increased MD in the hippocampus
Peng et al., 2017^51^	69 PD, 103 HC (from PPMI)	GM, WM, CSF volumes, cortical thickness, cortical surface area, correlation index of cortical thickness of 78 ROIs	Feature selection: Recursive feature elimination; Classification: SVM; Validation: tenfold cross-validation	Classification accuracy: 85.8% (combined all features), 71.6% (GM + WM + CSF)	The most sensitive features are in the frontal lobe, parental lobe, limbic lobe, temporal lobe, and central region
Amoroso et al., 2018^25^	374 PD 169 HC (from PPMI)	Network measures (correlation of patch voxel intensity distribution) from MRI images, and clinical data	Feature selection: Random forest; Classification: SVM; Validation: tenfold cross-validation	Classification accuracy (AUC): 0.88 ± 0.06 (MRI network measures); 0.70 ± 0.08 (clinical data); 0.93 ± 0.04 (combined features)	This MRI network approach has better classification accuracy than VBM (0.86 ± 0.06) and ROI (0.72 ± 0.07).
Singh et al., 2018^27^	408 PD 71 SWEDD (from PPMI); [128 AD 262 HC 447 MCI (from ADNI)]	Discretized Voxel Intensity Changes extracted from MRI images by SOM	Feature selection: SOM; Classification: SVM; Validation: tenfold cross-validation	Classification accuracy: 92.63 ± 0.06% (PD vs. HC); 94.63 ± 0.05% (PD vs. SWEDD); 92.65 ± 0.08% (SWEDD vs. HC); [94.29 ± 0.08% (AD vs. HC); 85.43 ± 0.08% (AD vs. MCI); 95.24 ± 0.05% (MCI vs. HC);]	Biomarkers were identified to further identify clinically relevant ROIs for differential diagnosis

*ADNI* Alzheimer’s disease neuroimaging initiative, *AUC* area under the receiver-operating characteristic (ROC) curve, *PD* Parkinson disease, *GM* gray matter, *WM* white matter, *CSF* cerebrospinal fluid, *FA* fractional anisotropy, *MD* mean diffusivity, *RD* radial diffusivity, *AD* axial diffusivity, *PSP* progressive supranuclear palsy, *MCI* mild cognitive impairment, *MSA* multisystem atrophy, *TBSS* tract-based spatial statistics, *VBM* voxel-based morphometry, *PCA* principal components analysis, *PPMI* Parkinson’s progression markers initiative, *SVM* support vector machine, *SWEDD* scans without evidence of dopaminergic deficit, *KSOM* Kohonen self-organizing map, *WAT* Welch–Aspin test, *UPDRS* unified Parkinson’s Disease Rating Scale, *GLM* generalized linear models, *MMRM* mixed-effect modeling with repeated measurements, *GEE* generalized estimating equations, *JFSS* joint-feature sample selection, *ReHo* regional homogeneity, *ALFF* amplitude of low-frequency fluctuation, *PIGD* postural instability and gait difficulty, *RBD* rapid eye movement (REM) sleep behavior disorder, *ROI* region of interest, *TD* tremor-dominant, *SOM* self-organizing maps.

**Table 4:** Machine-learning-based fMRI studies for PD diagnosis and early detection.

Study	Sample	Data	Methods	Main findings	Other findings
Long et al., 2012^30^	19 PD, 27 HC	fMRI: ALFF, ReHo, RFCS; MRI: volumes of GM, WM, CSF	Feature selection: 2-sample *t*-test; Classifier: SVM Validation: leave-one-out	PD showed decreased ALFF in ROL_L, decreased ReHo in bilateral ORBmid; increased RFCS in PHG_L, ANG_L, MTG_R; Classification accuracy: All modal: 87% (sensitivity: 79%; specificity: 93%)	PD showed increased GM in PCG, decreased GM in PCL_L and increased WM in regions such as PreCG_R; Classification accuracy: ReHo+ALFF + RFCS: 74%; ALFF + RFCS:67%; GM + WM + CSF: 80%;
Zhang et al., 2014^79^	25 PD (15-tremor, 10 non-tremor), 20 HC	Regional network efficiencies (i.e., the local and global efficiencies)	Feature selection: nonparametric permutation tests and *t*-test; Classifier: Maximum uncertainty linear discriminant analysis; Validation: leave-one-out	Regions distinguishing between PD and HC: the limbic system (e.g., bilateral hippocampus and thalamus), basal ganglia (e.g., bilateral caudate and left putamen), cerebellum, insula and cingular cortex; Classification accuracy (PD vs. HC): 89% (sensitivity 100%, specificity 80%)	Classification accuracy: (tremor-PD vs. HC) 97%; (non-tremor-PD vs. HC) 90%; (tremor-PD vs. non-tremor-PD) 92%
Chen et al., 2015^64^	21 PD, 26 HC	Network-based whole-brain FC	Feature selection: Kendall tau rank correlation coefficient comparison; Classifier: SVM; Validation: leave-one-out	The most discriminative FCs in: DMN, CO and FP networks and the cerebellum; Classification accuracy: 93.6% (sensitivity of 90.5% and a specificity of 96.2%)	Whole-brain functional connectivity might provide more information for discrimination than do any other characteristics (GM, WM, CSF, ALFF, ReHo and RFCS)
Gu et al., 2016^80^	52 PD (19 PIGD, 25 TD, 8 mixed subtype), 45 HC	GM, WM, CSF volumes from MRI; FA, MD, RD, AD from DTI; ReHo and ALFF from resting-state fMRI	Feature selection: Recursive feature elimination; Classification: SVM; Validation: Leave-one-out	Classification accuracy: PIGD vs. non-PIGD 92.3%	The diagnostic agreement evaluated by the Kappa test showed Kappa value = 0.83 for agreement with the existing clinical categorization
Herz et al., 2016^81^	12 PD with LID, 12 PD without LID	Seed-based FC in cortico-striatal network (between putamen and SMA, PSMC, and R IFG)	Feature selection: none; Classifier: SVM; ROC analysis; Linear regression analysis used to test whether FC could predict dyskinesia severity; Validation: leave-one-out	FC between putamen and PSMC increased after levodopa intake in No-LID pts and decreased in LID pts; Classification accuracy (LID vs. no-LID): 95.8% (91.7% Sensitivity;100% Specificity)	FC between putamen and PSMC predicted LID severity (R(2) = 0.627, P = .004); Volumes of putamen, PSMC or SMA did not distinguish LID from no-LID
Badea et al., 2017^62^	(1) NEUROCON: 27 PD, 16 HC; (2) PPMI: 91 PD, 18 HC; (3) Wu: 20 PD, 20 HC	FC obtained from ROI pairs (using parcellations such as Power 264 regions, Gordon 333 regions and Talairach 695 regions)	Feature selection: *t*-test; Classifier: SVM, Gaussian Naive Bayes; Validation: 50-fold cross-validation	Reproducibility of PD-related FC changes was low across the 3 datasets; Classification accuracy: 50~60% (trained and tested on the same dataset); <50% (trained on one dataset, tested on another)	Different parcellations revealed different FC decrease (between different ROI pairs) in PD
Pläschke et al., 2017^63^	80 PD, 95 HC(old), 93 HC(young), 86 SCZ	FC from 12 networks such as motor network	Feature selection: Log-likelihood ratios Classification: SVM; ROC analysis; Log-likelihood ratios; Validation: tenfold cross-validation	FC in motor network had the best discrimination power between PD and HC (followed by memory and cognition networks); Classification accuracy: 70% (AUC: 0.77)	FC in all 12 networks performed better in young-old classification than other classifications (highest single network AUC: 0.93); FC in emotion processing, empathy and cognitive action control networks differentiate SCZ from HC (highest single network AUC: 0.79)
Tang et al., 2017^56^	51 PD, 50 HC	ALFF, fALFF	Feature selection: *t*-test Classifier: SVM; Validation: leave-one-out	Altered ALFFs in the bilateral lingual gyrus and left putamen and an altered fALFF in the right posterior cerebellum; Classification accuracy: 84.2%; sensitivity 88.2%; specificity 80%	With un-optimized SVM classifier, the poorest classification performance was > 80%; Optimization of the classifier improved classification performance

*R* right, *L* left, *ALFF* amplitude of low-frequency fluctuations, *ANG_L* left-angular gyrus, *fALFF* functional ALFF, *LID* levodopa-induced dyskinesias, *MTG_R* right middle-temporal gyrus, *RFCS* regional FC strength, *ROL* rolandic operculum, *SMA* supplementary motor area, *mPFC* mesial prefrontal cortex, *PHG_L* left parahippocampal gyrus, *R MFC* right middle-frontal gyrus, *ROC* receiver-operating characteristic analysis, *PDRP* PD-related pattern, *DMN* default mode network, CO cingulo-opercular, *FP* frontal-parietal, *PPMI* Parkinson’s progression markers initiative, *PSMC* primary sensorimotor cortex, *IFG* inferior frontal gyrus.

## Machine-learning-based studies for the discrimination between PD and HC

### Dopaminergic imaging

Reduced uptake of a dopamine transporter radiotracer in the striatum of a PD patient on dopaminergic imaging (SPECT or PET) indicates neuronal degeneration and dopaminergic deficit in PD. In particular, reduced uptake of [^123^I]FP-CIT ([^123^I]-ioflupane) (the most widely used dopamine transporter radiotracer in SPECT DaTSCAN imaging) in the striatum (putamen and caudate) helps confirm PD and exclude other disorders such as drug-induced Parkinsonism and essential tremor. Measurements of dopamine transporter binding in the striatum and the distribution of the radiotracer uptake are important to characterize dopaminergic functional deficit in PD. Semi-quantitative analysis computes measurements of dopamine transporter binding in the striatum such as striatal uptake and striatal-binding ratios, but can not capture the distribution of the radiotracer uptake (which is often perceived by experienced experts), while machine-learning methods such as artificial neural network (ANN) and support vector machine (SVM) can identify data patterns in the distribution of the radiotracer uptake on SPECT imaging.

To test the ability whether a machine-learning method can mimic expert pattern recognition skills, Acton and Newberg applied ANN to striatum images obtained from dopaminergic SPECT imaging and obtained an overall diagnostic accuracy of 94.4% (*n* = 81)^15^. Illan et al.^44^ further developed an automatic computer-aided diagnostic system based on SVM (and other classifiers) for PD detection, and found that classification with SVM on striatum images performed the best (the area under the receiver-operating characteristics curve (AUC) was 0.968) (*n* = 108). In addition, Segovia et al.^45^ used partial least square (PLS) for data dimension reduction of striatum images, classified the imaging features with SVM and obtained a classification accuracy of 94.7% (*n* = 95). Further, Palumbo et al.^46^ used SVM to classify the uptake values in the striatal regions (accuracy: 90.6–90.7%) and reported that uptake values in the putamen are the most discriminative predictor for PD diagnosis, and adding patient age to data classification improved classification accuracy (95.6%) (*n* = 56).

Comparative studies between machine-learning-based analysis and semi-quantitative analysis of SPECT images have shown that computer-aided diagnosis (CAD) based on machine-learning methods such as SVM and ANN has outperformed conventional semi-quantitative analysis, reduced interpretation variability of dopaminergic transporter SPECT imaging, and improved diagnostic accuracy of PD and consistency of radiologists^15,19,20^.

Further, new imaging features such as texture features have improved classification accuracy (e.g., 97.4%, *n* = 158)^47^, and recently developed machine-learning techniques such as deep-learning convolutional neural networks (CNNs) have begun to show some promising results. For example, Choi et al.^18^ developed an automatic deep-learning system that applied CNNs to SPECT imaging analysis and obtained high detection rates of 96% (PPMI data, *n* = 431, early PD) and 98.8% (local data, *n* = 72, advanced PD), which was comparable to that of experts’ visual analysis and semi-quantitative analysis. The deep-learning system could also reclassify patients who were clinically diagnosed as PD, but had scans without evidence of dopaminergic deficit (SWEDD)^18^. Further, it has been reported that new classifiers such as enhanced probabilistic neural network and a semi-supervised-learning classifier graph-based transductive learning detected PD more accurately than SVM^37,48^. In addition, to overcome the limitations of institution-specific ML software implementations, Zhang and Kagen explored the widely available Google^TM^ TensorFlow machine-learning software library and applied Artificial Neural network to SPECT image classification for a large sample of PD patients (*n* = 1171), which yielded a classification accuracy of 93.8 ± 4.7%^49^. Further, Glaab et al.^21^ performed voxel-based whole-brain analysis on FDOPA PET (*n* = 60 PD) and FDG PET (*n* = 44 PD) images, classified them with SVM and random forest models, and found that FDOPA PET had (~10%) higher diagnostic performances than FDG PET. Using uptake features or texture features extracted from FDG PET data, classification yielded 70–91% accuracy^21–23^. Further research is warranted to validate and optimize these new data features and/or new machine-learning methods to improve classification accuracy and reliability.

### Structural magnetic resonance imaging (MRI)

Morphometric measurements such as brain gray matter (GM) and white matter (WM) volumes, shapes, cortical thickness, and cortical surface area in regions of interest (ROIs) such as striatum have been used as imaging features to detect progressive brain atrophy in machine-learning-based MRI imaging analysis to aid in the diagnosis of PD.

Subcortical nuclei shape analysis has revealed volume differences in the putamen and shape differences in the striatum (putamen and caudate nucleus) between PD patient group and control group, and discriminant analysis using a combination of these imaging features discriminated individual patients from controls with an accuracy of 75–83% (*n* = 21)^50^. In another study, SVM was applied to combined MRI imaging features (GM, WM, cerebrospinal fluid (CSF) volumes, cortical thickness, cortical surface area, correlation index of cortical thickness of 78 ROIs), which distinguished patients from controls with an accuracy of 85.8% (*n* = 69)^51^. In addition, new MRI imaging features such as cerebellum shape index^36^ or GM density feature of the cerebellum^52^ and proper classifiers are promising to improve classification accuracy. For example, classifying GM density decrease in the Crus and Vermis of the cerebellum with SVM improved the classification accuracy to 97%^52^, while classifying neuroimaging biomarkers such as cerebellum shape index, surface area, and volume of regions of interest (ROIs), as well as clinical data (e.g., UPDRS scores) with AdaBoost classifier, yielded a classification accuracy up to 98.9%^36^.

### Functional MRI (fMRI)

Reduced functional connectivity (FC) and brain activity (measured by amplitude of low-frequency fluctuation (ALFF)) in the basal ganglia network (BGN) and sensorimotor network in PD have been reported^53–56^. These findings are consistent across multiple patient samples^54,57^, and robust to variations in image processing methods, which suggests that resting-state fMRI (rs-fMRI) might be a biomarker for PD.

However, there are some inconsistent findings across rs-fMRI studies in PD^58–62^. Machine-learning methods may help reveal the diagnostic value of rs-fMRI in PD and clarify some of the inconsistencies. rs-fMRI measurements such as FC, ALFF, and regional homogeneity (ReHo) have been used as imaging features for PD classification. For example, classifying FC, ALFF, and ReHo features with SVM yielded a classification accuracy of 74% (*n* = 19)^30^; using FC features from 12 brain networks, FC classification with SVM yielded 70% accuracy (*n* = 80)^63^, while using whole-brain FC features, classification with SVM achieved 93.6% accuracy (*n* = 21)^64^. Apart from variations across data samples, these results revealed the importance of feature selection and optimization in machine-learning-based rs-fMRI image analysis.

### Multi-modal data

Since data from a single source or modality (e.g., SPECT) can not fully capture all the key characteristics of the abnormalities of PD, multi-modal data (e.g., combined SPECT imaging and clinical data such as motor test score) may help improve PD detection. Multi-modal data refers to data from different sources (such as imaging modalities: SPECT, PET, MRI, etc.; and/or clinical tests: motor test, cognitive test, etc.) measured on different scales. Clinical data that are often used for PD diagnosis include motor data and non-motor data of clinical examinations such as motor disorder society-sponsored revision of the Unified Parkinson’s Disease Rating Scale I, II, and III (MDS-UPDRS I,II, and III), Montreal Cognitive Assessment (MoCA), Scales for Outcomes in Parkinson’s Disease—Autonomic (SCOPA-AUT), and University of Pennsylvania Smell Identification Test. In addition, to identify biomarkers of PD progression, the Parkinson Progression Marker Initiative (PPMI)^5^, a comprehensive international multi-center study collected multi-modal clinical data (motor data included MDS-UPDRS; non-motor data included cognitive testing such as MoCA, autonomic testing such as SCOPA-AUT total autonomic score, sleep disorder assessment, and olfactory assessment), imaging data (DaTSCAN and MRI), biospecimen data (blood, CSF, urine) and genetic data (DNA, RNA) of 400 PD patients and 200 health controls over 5 years, and made the data available online, which is a great data resource in the field^5^.

Studies have shown that the combination of imaging and clinical data have improved the detection of brain abnormalities in PD^17,21,36,37^. For instance, Hirschauer et al.^37^ found that PD detection rate of a single-modal feature extracted from SPECT (Ioflupane (^123^I) striatal-binding ratios in the caudate and putamen) was 66–97%, but the combined multi-modal data features (SPECT + clinical data) yielded a detection rate of 98.6%. Glaab et al.^21^ also reported that combining imaging data features (PET data) with metabolomics data enhanced the discrimination power and diagnostic performance of the machine-learning systems in their study.

In addition, studies have shown that combined genetic and clinical data (such as rapid eye movement (REM) sleep behavior disorder, olfactory loss, and CSF measurements) improved PD diagnosis and early detection^17,36,37^. Genetic data (e.g., whether sibling with PD with age of onset <50 years) and clinical data such as abnormal quantitative motor test results are biomarkers of early PD^9^. For a recent review on machine learning using genetic data in PD, see ref. ^65^.

## Machine-learning-based studies for the differential diagnosis of PD

### Dopaminergic imaging

The difference in striatal uptake or striatal uptake ratios between PD and other parkinsonism has been identified by machine-learning-based dopaminergic imaging analysis. To differentiate between PD and vascular parkinsonism (VP), Huertas-Fernández et al.^66^ developed diagnostic models to classify the [(123)I]FP-CIT uptake in the region of interest (ROI) striatum and the whole brain, and reported that discrimination accuracy between VP and PD reached 90.3 ± 5.8% (using logistic regression for ROI approach), and 90.4 ± 5.9% (using SVM for voxel-based whole-brain approach) (*n* = 164). Further, to differentiate between PD and atypical parkinsonian syndromes such as MSA or PSP, SVM has been applied to (^18^)F-DMFP PET image classification (with imaging features such as striatal uptake and uptake in the thalamus) and has yielded moderate (>70%) classification accuracy (*n* = 39)^67–69^. A recent study has shown that using deep-learning method and saliency features (extracted from FDG-PET images) significantly improved the differentiation between PD, MSA, and PSM (*n* = 502)^24^.

In addition, attempts have been made to differentiate between PD and essential tremor with machine-learning-based dopaminergic imaging analysis. Using striatal uptake ratios as input data for ANN, Hamilton et al.^70^ distinguished PD from essential tremor (*n* = 18) with 100% diagnostic accuracy. Further, Palumbo et al.^71^ classified striatal uptake ratios with probabilistic neural network (PNN) (*n* = 261), and confirmed that PNN achieved valid classification accuracy to differentiate between PD and essential tremor (accuracy: 81.9 ± 8.1% for early PD; 78.9 ± 8.1% for advanced PD; 96.6 ± 2.6% for essential tremor).

### Structural MRI

Atrophy in the midbrain, basal ganglia, and cerebellar peduncles helps distinguish PD from atypical parkinsonian disorders such as progressive supranuclear palsy (PSP) and multiple system atrophy (MSA). PD has subtle volume reduction in cerebral gray matter (GM) and the basal ganglia^50,72,73^, while major brain atrophy of PSP is in the midbrain and superior cerebellar peduncles^2^, and for MSA, major abnormalities are in pons, middle cerebellar peduncles, and cerebellum^2,74^. Although challenging, attempts to use machine-learning approach have been made to differentiate between PD and other parkinsonian types based on these structural MRI imaging features.

To distinguish PD from atypical parkinsonian disorders (such as PSP or MSA), Duchesne et al.^75^ developed an automated computer classification system that extracted brain tissue composition and deformation features in the hindbrain region from MRI images, applied SVM to feature classification and obtained a classification accuracy of 91% (PD vs. non-PD (PSP or MSA)) (*n* = 16 PD). Further, to differentiate PD from atypical parkinsonian disorders, Focke et al.^76^ used GM and WM volumes obtained by voxel-based morphometry (VBM) and found that classification with SVM yielded up to 96.8% accuracy for differentiation between PD and PSP, and 71.9% between PD and MSA, but it failed to differentiate between PD and healthy controls (*n* = 21 PD). On the other hand, Salvatore et al.^77^ extracted MRI imaging features by principal components analysis, generated voxel-based pattern distribution map of structural differences for identification of voxel-based morphological biomarkers of PD and PSP, and obtained >90% accuracy in differentiating between PD and PSP, or between PSP and healthy control (*n* = 28 PD). To further distinguish PD from PSP and MSA, Huppertz et al.^74^ developed an automated MRI analysis method that computed atlas-based volumetric measures and classified the imaging features with SVM, reported the majority of classification accuracy of >80%, and found the largest atrophy in PD, PSP, and MSA (compared with controls) (*n* = 204 PD). To differentiate between PD and scans without evidence of dopaminergic deficit (SWEDD) or healthy controls, Singh et al.^26,27^ extracted discretized voxel intensity changes from MRI using unsupervised self-organizing maps, classified the imaging features with SVM (*n* = 408 PD) and achieved accurate classification performances (>90%).

In addition, abnormalities in the substantia nigra in PD (due to dopaminergic neuronal loss) revealed by T2-weighted MRI, neuromelanin-sensitive MRI or iron-sensitive MRI at high field strength (such as 7 T) or by 3 T susceptibility weighted imaging (SWI) can be used in the diagnosis of PD^14^. For example, Haller et al.^78^ examined PD patients with SWI and found that they had increased SWI in the bilateral thalamus and left substantia nigra, which had diagnostic value in differentiating between PD and other parkinsonism (classification accuracy for SVM: 86.92 ± 16.59%) (*n* = 20 PD).

### Functional MRI (fMRI)

Machine-learning methods have also been applied to rs-fMRI analysis for differentiation of PD subtypes such as tremor-PD vs. non-tremor-PD^79^, and postural instability and gait difficulty subtype (PIGD) vs. non-PIGD^80^. Zhang et al.^79^ used rs-fMRI measurement regional network efficiencies as imaging feature, and classified them with linear discriminant analysis, which yielded an accuracy of 92% in differentiating tremor-PD vs. non-tremor-PD^79^. Further, to differentiate between PD with Levodopa-induced dyskinesias (LID) and PD without LID, Herz et al.^81^ extracted seed-based FC in cortico-striatal network from rs-fMRI images, classified them with SVM and achieved a differentiation accuracy of 95.8%.

### Diffusion tenser imaging (DTI)

Reduced substantia nigra fractional anisotropy (FA) has been identified and regarded as a PD biomarker for over a decade, but recent meta-analyses have found that substantia nigra fractional anisotropy had a very large variation in results across studies^82^, had low pooled sensitivity and specificity, and was not a diagnostic biomarker of Parkinson’s disease^83^.

However, since DTI reflects the disruption of microstructure (e.g., neuron myelin) integrity, DTI has shown promise in differentiating PD from atypical parkinsonism. Haller et al.^84^ examined DTI images of PD patients and other Parkinsonism with tract-based spatial statistics (TBSS) analysis and found that compared with other parkinsonism, PD patients had an increased FA and a decreased MD in the right frontal white matter, and classification of DTI imaging features using SVM yielded an accuracy of 97.5 ± 7.54% (*n* = 17 PD vs. 23 other Parkinsonism). Further, Cherubini et al.^85^ combined DTI and MRI voxel-based morphometry features to distinguish PD patients from PSP patients using SVM, which yielded an improved accuracy (100%) (*n* = 57 PD vs. 21 PSP). Combining with MRI voxel-based morphometry and rs-fMRI imaging features, DTI has also aided in the differentiation between PD subtypes (PIGD vs. non-PIGD)^80^. In addition, using combined DTI and apparent transverse relaxation rate (R2*) imaging, Du et al.^86^ found that MSA has a decreased FA and an increased apparent transverse relaxation rate (R2*) in the subthalamic nucleus, whereas PSP has an increased MD in the hippocampus. Classification of imaging features with Elastic-Net machine-learning technique yielded high differentiation accuracy (>90%) (*n* = 35 PD vs. 16 MSA vs. 19 PSP)^86^.

### Multi-modal data

Compared with single-modal data features, higher classification accuracy or detection rate using combined multi-modal data features has been achieved in differentiation between PD and atypical parkinsonian disorder. For instance, to differentiate between PD and PSP, Cherubini et al.^85^ used combined MRI and DTI imaging features, and achieved a classification accuracy of 100%, which was higher than using either MRI or DTI features alone^85^. In addition, to differentiate between PD and MSA or PSP, Du et al.^86^ reported high classification accuracy (98–99%) using DTI and apparent transverse relaxation rate R2* imaging features, higher than DTI or R2* features alone.

## Machine-learning-based studies for the early detection of PD

### Dopaminergic imaging

Machine learning has been found useful in dopaminergic imaging analysis for early PD detection. As a pioneering study, Prashanth et al.^16^ investigated the value of different SVM methods in classifying SPECT images for early PD detection. Using striatal-binding ratios in the striatal regions from data obtained from the PPMI database, they found that SVM was valuable in early PD detection and SVM with non-linear kernel achieved higher detection rate (96.14 ± 1.89%) than SVM with linear kernel. Oliveira et al.^87^, applied SVM and other classifiers to classification of the binding potential at each voxel in the striatum of SPECT images for early PD detection and reported that SVM achieved the highest detection rate (97.86%). Later, Prashanth et al.^17^ added non-motor clinical data features such as cerebrospinal fluid (CSF) measurements to further improve the detection rate of early PD. Prashanth et al.^88^ further found that shape and surface-fitting-based features showed higher importance than striatal-binding ratios for early PD detection and feature classification with SVM yielded a classification accuracy of 97.29 ± 0.11% (*n* = 427)^88^. In addition, Oliveira et al.^89^ found that the length of the striatal region uptake (detection rate: 96.5%) performed better than uptake ratio-based based features for early PD detection (*n* = 443). These findings have demonstrated the value of machine-learning approach in dopaminergic image analysis for early detection of PD and the importance of imaging feature and classifier selection/optimization in machine-learning-based imaging analysis.

### Structural MRI

Machine-learning methods have helped improve the diagnostic gain of MRI. Classification of combined MRI measurements (gray matter (GM) + white matter (WM) + cerebrospinal fluid (CSF) volumes) with SVM could detect early PD with an accuracy of 80% (*n* = 19)^30^. A robust linear discriminant analysis (LDA) classifier with an optimal set of imaging features (GM and WM volumes of 98 ROIs from MRI) that applied to MRI images of early PD patients (99% in early stages) (*n* = 374) yielded a classification accuracy of 81.9%, which was higher than 69.1% yielded by SVM^90^. In addition, Singh and Samavedham^26^ demonstrated that the structural changes of early PD could be detected by using MRI features alone with an unsupervised self-organizing map approach (*n* = 518) and high classification accuracy (>95%) was achieved. This approach was later applied to a larger dataset (*n* = 1316) from PPMI (Parkinson’s Progression Markers Initiative) and ADNI (Alzheimer’s disease neuroimaging initiative), and yielded high classification performance (95.37 ± 0.02%) for distinguishing patients with PD (or Alzheimer’s disease) from healthy subjects^27^, which confirmed the value of the machine-learning approach in aiding the diagnosis of neurodegenerative disorders such as PD and Alzheimer’s disease. In a recent study, Amoroso et al.^25^ used an unsupervised approach for MRI image classification, and they extracted structure regional connectivity features from MRI images (n = 374), applied SVM to imaging feature classification, and obtained a classification accuracy of 88 ± 6% (using MRI features alone) or 93 ± 4% (using MRI features and clinical data).

### Functional MRI (fMRI)

Studies have shown that rs-fMRI can detect PD at early stages. Wu et al.^29^ used effective connectivity extracted from rs-fMRI to examine patients with early PD (*n* = 16) and reported that the substantia nigra pars compacta in early PD had decreased effective connectivity with regions such as the striatum, thalamus, supplementary motor area and cerebellum, which negatively correlated with the Unified Parkinson’s Disease Rating Scale (UPDRS) scores. However, the detection rate of early PD using rs-fMRI imaging features alone (classified by SVM) is not high (e.g., 74%^30^) and needs to be improved. In addition, the findings of two rs-fMRI studies in asymptomatic LRRK2 mutation carriers suggested that functional connectivity disruptions precede the presence of PD motor symptoms^32,33^. Further, using rs-fMRI, Rolinski et al.^31^ examined patients with rapid eye movement (REM) sleep behavior disorder (RBD) (*n* = 26), and PD patients (*n* = 10), and found that functional connectivity measures of basal ganglia network (BGN) dysfunction differentiated RBD and PD from HC with high sensitivity (96%) and specificity (74% for RBD, 78% for PD), suggesting that rs-fMRI may be a biomarker in identifying early functional connectivity changes in the BGN in subjects at high risk of PD and patients with PD. However, confirmative studies are warranted.

### Multi-modal data

Combining multi-modal imaging and/or clinical data have improved early PD detection. Long et al.^30^ found that combined multi-modal features improved early PD detection and multi-modal imaging (MRI and rs-fMRI) with combined multi-modal features (GM + WM + CSF + ReHo+ALFF + FC) yielded higher classification accuracy (87%) than single-modal features (MRI: 80%; rs-fMRI:74%). In addition, Oliveira et al.^89^ examined SPECT images of early PD patients and found that several data features had high classification accuracy including the length of the striatal region (96.5%), the putaminal binding potential (95.4%) and the striatal-binding potential (93.9%), while the combined imaging features had the highest classification accuracy (97.9%). Furthermore, Prashanth et al.^17^ classified non-motor clinical data features, such as rapid eye movement (REM) sleep behavior disorder (RBD) and olfactory loss, and CSF measurements in addition to SPECT imaging markers (striatal-binding ratios) with classifiers such as SVM and random forests, and found that a combination of these data features with SVM classification performed the best in early PD detection (detection rate: 96.40 ± 1.08%) (*n* = 401).

## Discussion

The studies reviewed in this paper have demonstrated that machine-learning automated data analysis, identified data patterns (e.g., in the distribution of the radiotracer uptake on SPECT images) and improved the accuracy of imaging quantification in the diagnosis of PD. A recent comprehensive review has confirmed the value of machine learning in assisting the diagnosis of PD, and has further pointed out the potential of these machine-learning applications to enhance clinical decision-making in PD diagnosis^91^. Particularly, the review by Mei et al.^91^ provided statistical analysis for the machine-learning studies in PD diagnosis, and reported that (1) on average, the classification accuracy of the machine-learning applications was ~94% for SPECT imaging, ~86% for PET imaging, and ~87% for MRI (including fMRI) imaging; (2) SVM and NN (neural network) were the most frequently used methods in the imaging studies, the usage for SVM (50%–70% used for SPECT or PET imaging, ~60% for MRI imaging) was higher than that of NN (22%–53% used for SPECT or PET imaging, ~23% for MRI imaging); and (3) SVM and NN had higher classification accuracy than other machine-learning methods in the imaging studies.

### The value and role of machine learning in the diagnosis and early detection of PD

The value and potential of machine learning (ML) in PD diagnosis have been clearly demonstrated by comparative studies that compared ML methods with conventional techniques such as semi-quantitative methods or visual analysis in the diagnosis of PD. For example, it has been shown that computer-aided diagnosis (CAD) system based on machine-learning methods has outperformed semi-quantitative methods of SPECT image analysis^15,19^ and improved PD diagnostic accuracy of radiologists^20^. Further, Choi et al.^18^ demonstrated the value of recently developed deep-learning techniques (convolutional neural networks) in SPECT image analysis, and obtained high classification performance that is comparable to experts’ visual analysis and semi-quantitative analysis^18^. In addition, ML methods have been shown useful in differential diagnosis^65–69,85,86^ and early PD detection^17,26,30,31^.

Machine-learning applications can automatically analyze and classify imaging and clinical data in PD, but machine-learning applications are still in infancy and subject to errors, pitfalls and biases. For example, clinician-dependent class/group labeling of the training data in the machine-learning models may be prone to errors because of the high error rate in the clinical diagnosis of PD. As another example, newly developed deep-learning models may have new challenges such as overfitting, low generalizability and data insufficiency.

The role of machine-learning applications is not to substitute clinicians, but to assist them in clinical decision-making, to relieve them from tedious data preprocessing, to save them from time-consuming manual raw data inspection or processing (e.g., draw regions of interest and perform measurements on images), and to help them focus on important clinical decision-making questions in order to reduce medical errors, and improve the clinical diagnosis of PD. On the other hand, machine-learning applications are far from perfect and still need to be improved. Being aware of the potential errors and problems in machine-learning applications in radiology, Geis et al.^92^ pointed out that clinicians who use the applications are ultimately responsible for clinical decision-making and patient care.

### Current limitations and challenges in machine-learning applications

#### First, prone-to-error labeling of classes/groups in supervised learning

Due to the high error rate of a clinical diagnosis of PD, clinician-dependent labeling for the classes or groups (e.g., PD patients or healthy subjects) of the training data (that are used in supervised machine-learning applications) may be prone to error. To overcome this problem, training data labeling in supervised learning need to be confirmed by pathological (biopsy or post-mortem) data. On the other hand, when the clinical diagnosis of a PD dataset is uncertain and pathological data is not available, unsupervised-learning approaches may be considered. Without the need for training data, unsupervised learning seeks to identify hidden data patterns, which may overcome the problem of mislabeling diagnostic categories in the training data in supervised learning. However, there are some technical challenges in applying unsupervised-learning methods to imaging analysis in PD diagnosis, e.g., unsupervised-learning methods are not good at accurately extracting imaging features^43^. Attempts have been made to overcome such difficulties, e.g., by using semi-supervised-learning clustering method that combines a small amount of labeled data with a large amount of unlabeled data in the training dataset^43^. In addition, unsupervised learning has been applied to MRI feature selection in early PD detection^25–27^. To detect early PD using supervised-learning methods on structural MRI features is challenging, but Singh and Samavedham have demonstrated that the structural changes of early PD could be detected by integrating Kohonen unsupervised self-organizing map and least-squares support vector machine (*n* = 518)^26^. This approach was later applied to a larger dataset (*n* = 1316)^27^, which confirmed the value and robustness of the method.

#### Second, machine-learning “black-box”

Since machine-learning applications identify data patterns (e.g., abnormal structural or functional changes in imaging data) that could be invisible (or unrecognizable) to humans, the mechanisms and results of the machine-learning applications (especially for neural network-based models) may be difficult to interpret due to lack of direct “evidence” supporting classification results. This could be against the principle of evidence-based medicine and results in reluctance to accept machine-learning applications in clinical practice. For example, new deep-learning (or deep-neural network) models often have millions of parameters which make them like a “black-box” incomprehensible to clinicians, and make it hard to interpret how the classification results have been obtained. However, just like a microscope allows people to see at cellular level (which is invisible to human eyes), machine-learning methods identify abstract imaging features (that reveal brain signal differences between groups/classes) such as distributions of a radiotracer uptake, image voxel intensity changes, and texture features, and amplify these signal differences between classes/groups at a resolution that the signal differences between classes/groups can be detected by “machines” or machine-learning models to best separate the data into classes or groups. Clinicians do not have to understand the details of the inner workings (e.g., the parameters) of machine-learning methods in order to use these application tools for PD diagnosis, but it is beneficial to have some basic knowledge of the mechanism of a machine-learning method and statistical pitfalls in order to avoid errors. Nevertheless, although there is an abstraction in the mechanism of machine-learning methods, machine-learning applications shall follow the principle of evidence-based medicine, use best evidence in the field of PD diagnosis (e.g., to guide feature selection or check classification results), and provide as much “evidence” as possible to support clinical decision-making. For instance, in addition to imaging feature classification, Singh et al.^27^ identified disease-specific biomarkers (i.e., significant brain regions affected by PD) with a machine-learning method, and these biomarkers, serving as “evidence”, could be used to decipher disease progression. Further, efforts have been made to interpret classification results of deep-learning models. For example, Magesh et al.^93^ reported a newly developed deep-learning model CNN based on transfer learning to analyze and classify SPECT DaTSCAN images that could distinguish PD patients from healthy controls with an accuracy of 95.2%, and used Local Interpretable Model-Agnostic Explainer methods to interpret classification results.

#### Third, overfitting problem in machine learning

Overfitting problem often occurs in machine-learning applications, which refers to a machine-learning method or model performs very well on a training dataset, but not on a test dataset or other datasets^94^. This might be because the machine-learning method is over-trained by the training dataset and the noise in the training data is also modeled which makes the model ungeneralizable to other datasets. A recent rs-fMRI study showed that PD-related functional connectivity changes were not reproducible across the 3 PD samples used in the study^62^. The classification performance (PD vs. HC) was low (50–60%) even in the datasets from a single data sample and the lack of generalizability in these data samples may be mainly due to high PD heterogeneity^62^. In addition, there are new challenges of overfitting in machine-learning applications using deep-learning models^95^. To overcome the overfitting problem in machine-learning applications for PD diagnosis, it is necessary to improve data quality, reduce data heterogeneity, use large data samples and validate machine-learning models with proper validation methods (such as N-fold cross-validation) in order to make machine-learning models generalizable. Further, to avoid overfitting in deep-learning models, some methods such as implicit regulation, proper initiation, adjusting learning rates and reducing model complexity may help the models generalize well^95^.

### Future directions

#### First, improve and validate the machine-learning applications

Despite the progress made in machine-learning applications in the diagnosis and early detection of PD, there is still much room for improvement. 1) more research is needed to address the problem of prone-to-error class labeling in supervised learning. 2) it is necessary to optimize multi-modal data features for an optimal feature set, and choose and optimize machine-learning classifiers for an optimal classifier to improve classification accuracy. This is because: (1) several studies have shown that combined multi-modal data features (such as SPECT + clinical data) had higher detection rate than single-modal features^30,37^; (2) comparative studies using different classifiers have demonstrated the differences in classification accuracy between different classifiers^36,37,89^. 3) thorough validation is needed before the machine-learning applications can be used in clinical settings. In addition, newly developed deep-learning techniques have shown promising results^18,93,96^, but also face new challenges and obstacles such as overfitting, low generalizability and data insufficiency. For a recent review of deep-learning applications for the diagnosis of PD, see ref. ^97^. Research in explainable machine-learning models is needed to address the “black-box” problem in the neural network models. To overcome the new challenges in deep-learning models, further research is needed to avoid overfitting in deep-learning models, improve these new deep-neural network applications and make them more accurate, reliable, generalizable and explainable.

#### Second, improve modeling longitudinal multi-modal data

Since Parkinson’s disease is a progressive disorder, it is necessary to model multi-modal data over time in order to identify biomarkers for PD progression. Some efforts have been made to tackle this difficult problem in recent years^98–102^. Due to the complexity of longitudinal multi-modal (imaging and clinical) data, methods such as embedding learning and sparse regression have been proposed, which have obtained promising results^102^. Further research is needed to improve modeling these longitudinal multi-modal data so that reliable biomarkers can be identified to enhance the diagnosis and management of PD.

#### Third, integrate ML-based applications into clinical decision support system to aid in PD diagnosis

It has been demonstrated that the performance of machine-learning-based computer-aided diagnostic (CAD) system generally exceeded that of semi-quantitative analysis on SPECT imaging in distinguishing PD patients from healthy controls^12,16^ and improved PD diagnostic accuracy of radiologists^17^. More comparative and confirmative studies are needed to further reveal the advantages and weaknesses of these machine-learning applications. Since semi-quantitative imaging analysis software is commercially available at clinics, such software may be upgraded to incorporate mature machine-learning algorisms to further assist clinicians in the diagnosis of PD. However, a framework that facilitates the development, deployment, validation and regulation of such machine-learning-based clinical applications is needed. For example, benchmark data and metrics (e.g., the PPMI database) need to be established to test the optimized and standardized applications. Further, before these ML-based clinical applications are deployed in clinical settings, it is necessary to run clinical trials to assess the diagnostic gain and clinical benefits of such applications over conventional semi-quantitative analysis (or visual analysis). Consequently, rules and regulations are needed to facilitate this process in order to make such ML-based systems available to clinics.

## Conclusions

In summary, encouraging progress has been made in applying machine-learning techniques to the diagnosis and early detection of PD. Although machine-learning applications in PD diagnosis are still in their infancy, machine-learning methods have automated imaging data analysis, outperformed conventional semi-quantitative analysis and performed comparably well as experts’ visual inspection in detecting PD-associated dopaminergic degeneration on SPECT imaging, reduced interpretation variability of imaging, improved PD diagnostic accuracy of radiologists and aided in differential diagnosis and early PD detection. Using combined multi-modal imaging and clinical data (in these applications) may further enhance the diagnosis and early detection of PD. To integrate these machine-learning applications into clinical systems, further validation and optimization are needed to make them accurate and reliable. Despite the challenges in translating machine-learning applications into clinical practice, machine-learning techniques are promising to assist clinicians in improving differential diagnosis of parkinsonism and early diagnosis of PD, which may reduce the error rate of PD diagnosis, and help detect PD at pre-motor stage so that early treatments (e.g., neuroprotective treatment) may be applied to slow down PD progression, prevent severe motor symptoms from emerging, and relieve patients from suffering.

## Data Availability

Data sharing is not applicable to this article because this article is a literature review and no new data were created or analyzed in this study.
